# Asinine milk mitigates stress-mediated immune, cortisol and behavioral responses of piglets to weaning: A study to foster future interventions in humans

**DOI:** 10.3389/fimmu.2023.1139249

**Published:** 2023-04-14

**Authors:** Sharacely de Souza Farias, Ana Carolina Dierings, Vinicius Cardoso Mufalo, Leandro Sabei, Marisol Parada Sarmiento, Arthur Nery da Silva, Priscila Assis Ferraz, Guilherme Pugliesi, Claudio Vaz Di Mambro Ribeiro, Chiara Albano de Araujo Oliveira, Adroaldo José Zanella

**Affiliations:** ^1^ Department of Preventive Veterinary Medicine and Animal Health, School of Veterinary Medicine and Animal Science, University of São Paulo, Pirassununga, São Paulo, Brazil; ^2^ Faculty of Veterinary Medicine, University of Teramo, Teramo, Italy; ^3^ Department of Animal Reproduction, School of Veterinary Medicine and Animal Science, University of São Paulo, Pirassununga, São Paulo, Brazil; ^4^ Department of Animal Science, School of Veterinary Medicine and Animal Science, Federal University of Bahia, Salvador, Brazil; ^5^ Department of Preventive Veterinary Medicine and Animal Production, School of Veterinary Medicine and Animal Science, Federal University of Bahia, Salvador, Brazil

**Keywords:** nutraceutical profile, early-life stress, gut-brain axis, inflammatory cytokine, health

## Abstract

**Introduction:**

The present study assessed whether asinine milk supplementation improved the immune and behavioral responses of piglets during an early life weaning stress event as a model for its future use in humans.

**Methods:**

For this, 48 piglets from 4 different litters were used. At 20 days of age, piglets were weighed and allocated with their litter and dam into group pens until 28 days of age. Four piglets from each litter were then randomly assigned to either (1) asinine milk supplementation (n = 16) (2), skimmed cow milk supplementation (n = 16) or (3) no supplementation (n = 16; control group). The supplementations were voluntarily administered for 3 days preweaning and 3 days postweaning using a baby bottle. The effects on the weaning stress response were assessed through salivary cortisol measurements; behavioral tests such as the open field, novel object end elevated plus maze tests; and gene expression of HSD11B1, NR3C1 and IL1B in PBMCs, which was determined by RT−qPCR and normalized to GAPDH and UBB. To test the effect of the supplementations on weight, milk intake, gene expression, and behavior, a randomized block design was used with repeated measurements over time by the PROC MIXED procedure.

**Results and discussion:**

The effects on salivary cortisol were determined using the ratio between the morning and afternoon concentrations, considering the time before and after the weaning event. Principal component analysis (PCA) and Fisher’s test were performed to evaluate the behavior test data. When comparing salivary cortisol concentrations between the pre- and postweaning periods, there was a difference (p < 0.05) between the supplementation groups in the afternoon period, suggesting that piglets fed asinine milk had lower afternoon cortisol concentrations postweaning than their counterparts. For the behavioral tests, the supplementations had no measurable effects. No difference was between groups pre- and postweaning for the expression of HSD11B2, which codes for an enzyme that breaks down cortisol. However, the expression of NR3C1, which encodes the glucocorticoid receptor, was significantly upregulated in piglets supplemented with cow milk (mean 1.245; p < 0.05).

**Conclusion:**

Asinine milk downregulated 1L1B gene expression, which codes for an inflammatory cytokine. In conclusion, these results suggest that supplementation with asinine milk may represent a strategy to diminish the damage associated with an early life event by modulating IL1B expression and reducing salivary cortisol levels in piglets undergoing weaning stress. Further transcriptomic and metabolomic studies may improve our understanding of the molecular pathways that mediate this systemic immune-mediated response.

## 1 Introduction

Early life is a critical developmental window marked by vulnerability to permanent physiological and behavioral modulations (1–3), which may be induced by factors such as exposure to stress, the presence of pathogens and interactions with other individuals (4).

The effects of early life stressors may be alleviated with specialized nutrition, even for particularly susceptible individuals (5–7), for example, by administering vitamin B9, methionine, choline, betaine (5, 8, 9), folic acid (10–13) and polyunsaturated fatty acids (PUFAs – omega-3 and omega-6 FAs) (14–18). Some of these nutrients may interact with gene expression and the regulation of physiological mechanisms, thus affecting lifelong physical and mental health (3, 5). However, well-defined nutritional guidelines for specific categories of infants are still lacking (19, 20) and the administration of inadequate diets may exert long-lasting harmful effects (1, 21, 22).

The characterization of vulnerable developmental periods and the effects of possible interventions may be carried out with animal models in translational biology studies. In these translational studies, pigs are notable (23–25) for their similarities to humans in brain anatomy, neurodevelopmental processes (26, 27) and immune response (28–30). In piglets, a critical period of heightened sensibility to stressors, exposure to pathogens and intensified interactions with conspecifics is weaning. Within traditional systems, weaning is done abruptly between 21 and 28 days of age (31) and represents a significant challenge to piglets as it culminates in the end of maternal care (32, 33), changes in food source (31), exposure to new individuals and environments (34, 35), exposure to pathogens and procedures such as vaccination and administration of medication (32, 34, 36).

The combination of these factors results in significant stress, activating the hypothalamus-pituitary-adrenal (HPA) axis and stimulating the secretion of corticotropin releasing hormone (CRH), adrenocorticotropic hormone (ACTH) (37, 38) and glucocorticoid release (39, 40). Among glucocorticoid hormones, the most important indicator of stress is cortisol (41). Cortisol action is mediated by binding to glucocorticoid receptors, such as those encoded by the *NR3C1* gene (42–44).

Research has found that dietary characteristics are likely to coordinate the metabolic, endocrine, and immune functions of the host (45, 46), under basal and stressful circumstances. In one of the established mechanisms, research suggests that microbes communicate with the host, resulting in the production of hormones and consequent systemic responses (46). Increased levels of circulating cortisol are known to lead to long-lasting changes in metabolism, including a decrease in the host’s immune capacity to fight pathogens (47). These events may lead to temporary or permanent modulations in physiology, nervous system functioning, behavior, and immunity in young individuals (48–50).

Modulation of the immune system has lifelong consequences and may be studied through molecular biology assays, which are tools to explore gene expression levels and regulation methods (51). Recently, studies have focused on the abundance of mRNAs from peripheral blood mononuclear cells (PBMCs) (52) isolated from immune cells in whole blood (53), to assess gene expression changes in response to adverse stimuli associated with stressful events (54, 55).

The mRNA levels of the IL-1β, IL-6, and TNF-α genes measured in PBMCs can be used to assess the effects of weaning stress on the immune system. IL-1β encodes the IL-1β proinflammatory cytokine, which affects cells and organs and is an important mediator of several immunity-related disorders (56–59). It may also affect the production of glucocorticoids by binding to receptors in the hypothalamus as well act as agonists of the IL-1 receptor in the vagus nerve, increasing cortisol production in the adrenal cortex and causing the release of glucocorticoids into the bloodstream followed by a decrease in the concentrations of mRNA, decreasing its transcription and increasing gene destabilization (60). In addition, *NR3C1* and *HSD11B2* gene expression measurement in PBMCs can provide information on the impact of stress and treatment on the glucocorticoid receptor and glucocorticoid metabolism, respectively. The *NR3C1* and *HSD11B2* genes, which encode the glucocorticoid receptor and the 11 β-hydroxysteroid dehydrogenase type 2 enzyme, are involved in HPA-axis-mediated stress responses and their dysfunctions (61–63) and may reflect the effects of challenges such as weaning.

The responses are interrelated, as glucocorticoids also block posttranscriptional synthesis *via* cAMP and inhibit the release into the extracellular fluid, thereby decreasing the presence of inflammatory cytokines (64). Previous research has demonstrated that gene expression is also upregulated in piglets (55, 65), and calves (66, 67) undergoing weaning stress.

The physiological cascade associated with stress responses affects behavioral responses, including those associated with affective states. Regarding the study of affective states, there are several methods to assess them that have been validated in pigs (68). Behaviors considered to be indicators of anxiety and fear are the most studied in pigs (69) however, it is important to consider species-specific responses and mechanisms developed through evolution or selection (70, 71).

Dietary characteristics are capable of influencing and coordinating metabolic, endocrine, and immune functions under basal and stressful circumstances (45, 46), and translational studies between animals and humans are necessary to better understand the relationship and interactions between nutrition, the gut microbiota, neural physiology, and mental health, as well as the potentially modulatory effects of nutraceuticals particularly under stressful situations. One such potential nutraceutical is asinine milk, which has a high concentration of bioactive molecules and it is used to aid individuals with immunodeficiency and cardiac and psychological diseases (72–76). Asinine milk has probiotic potential, as it is rich in lactose, lysozyme (55, 68, 77) and PUFAs (78). Lysozyme exerts selective action on the gut microbiota (76, 79, 80). PUFAs are found in high concentrations in brain tissues and influence perceptive, intellectual and communication functions as well as growth and developmental processes (81). The ingestion of foods with increased fatty acid profiles (82) antioxidant capacity (28, 29) and probiotic activity (82, 83) may positively impact the prevention and control of neurologic disorders and ensure adequate growth and development of brain functions.

Asinine milk production is lower when compared to other dairy animals, asinine milk has nutraceutical properties; its therapeutic use has been described before (73, 84). Worldwide research aims to evaluate the properties of asinine milk and its use in human nutrition. Italy, China, Greece, France, and Kenya have become focal research points for asinine milk characteristics and nutritional quality (73, 85). Asinine milk can be used for therapeutical purposes, such as a complementary treatment option for tuberculosis, gastric ulcers, and metabolic diseases (86). to control coughing and pneumonia or prevent diseases (such as colds) in newborn children (87). In studies carried out with the elderly, the ability of donkey milk to modulate immune functions with a short administration time was proven (88).

Asinine milk possesses a nutraceutical profile, and weanling piglets are physiologically, emotionally and immunologically challenged. Therefore, the goal of this study was to assess whether asinine milk supplementation has the potential to improve immune and behavioral responses during an early life stress event in pigs as a model for future interventions in humans.

The effects of supplementation with asinine milk and skimmed cow milk on the weaning stress response were assessed through salivary cortisol measurements, behavioral tests, and mRNA quantification in plasma PBMCs from blood samples.

## 2 Materials and methods

### 2.1 Location and ethics statement

The study was conducted in the Department of Preventive Veterinary Medicine and Animal Health (VPS) (–21,9484694, –47,4563268) of the School of Veterinary Medicine and Animal Science of the University of São Paulo (FMVZ/USP), in the city of Pirassununga, state of São Paulo, Brazil. Field data collection was carried out between May and June of 2021, and laboratory and behavioral test analyses were performed between July and November of 2021. All procedures described in this study were approved by the Committee for the Use and Care of Animals in Research (CEUA) of the School of Veterinary Medicine and Animal Science of the University of São Paulo, protocol n° 8696141117 (ID 007216).

### 2.2 Animals and housing conditions

Four lactating sows and their litters were chosen for the study. The criteria for inclusion of animals in the experiment included a minimum number of 12 piglets per litter and a minimum age of 20 days. In total, 48 piglets from the TopGen Aphrodite^®^ lineage (Large White x Landrace) were used.

Sows and respective piglets were weighed and identified with nontoxic animal markers prior to transportation to the experimental facilities. Random numbers were assigned to the piglets at weighing, which were used throughout the experiment to distribute them into supplementation groups. All animals were then transferred to the experimental facilities.

Sows were housed with only their respective piglets in pens fitted with a heat lamp and rubber floor mats and were given hay as enrichment material during the ten days of the experiment. The first three days of housing were considered an adaptation period, and no experimental procedures were carried out. After this period, the experiment began with saliva samplings, behavioral tests, blood collection, milk supplementation, and weaning, followed by another round of saliva samplings, behavioral tests, and blood collection.

Piglets remained with their sows until 28 days of age, after which the sows were removed from the pens, and the piglets remained there until 30 days of age.

While sows and piglets were housed together, the sows received 7 kg of specialized lactation feed daily. No feed was offered to piglets while they were still nursing. Piglets only began receiving ad libitum nursery feed after weaning, and this feed contained 18% protein, 0.95% lysine and 3,300 kcal DE/kg, as per usual nutrition guidelines (89). Feed for both sows and piglets was distributed at 07:30 and 18:30 daily. All feed was produced at the University of São Paulo. Water was supplied ad libitum *via* nipple drinkers.

### 2.3 Experimental design, nutritional characteristics of the supplementation and management practices applied to the animals

**Figure 1:**
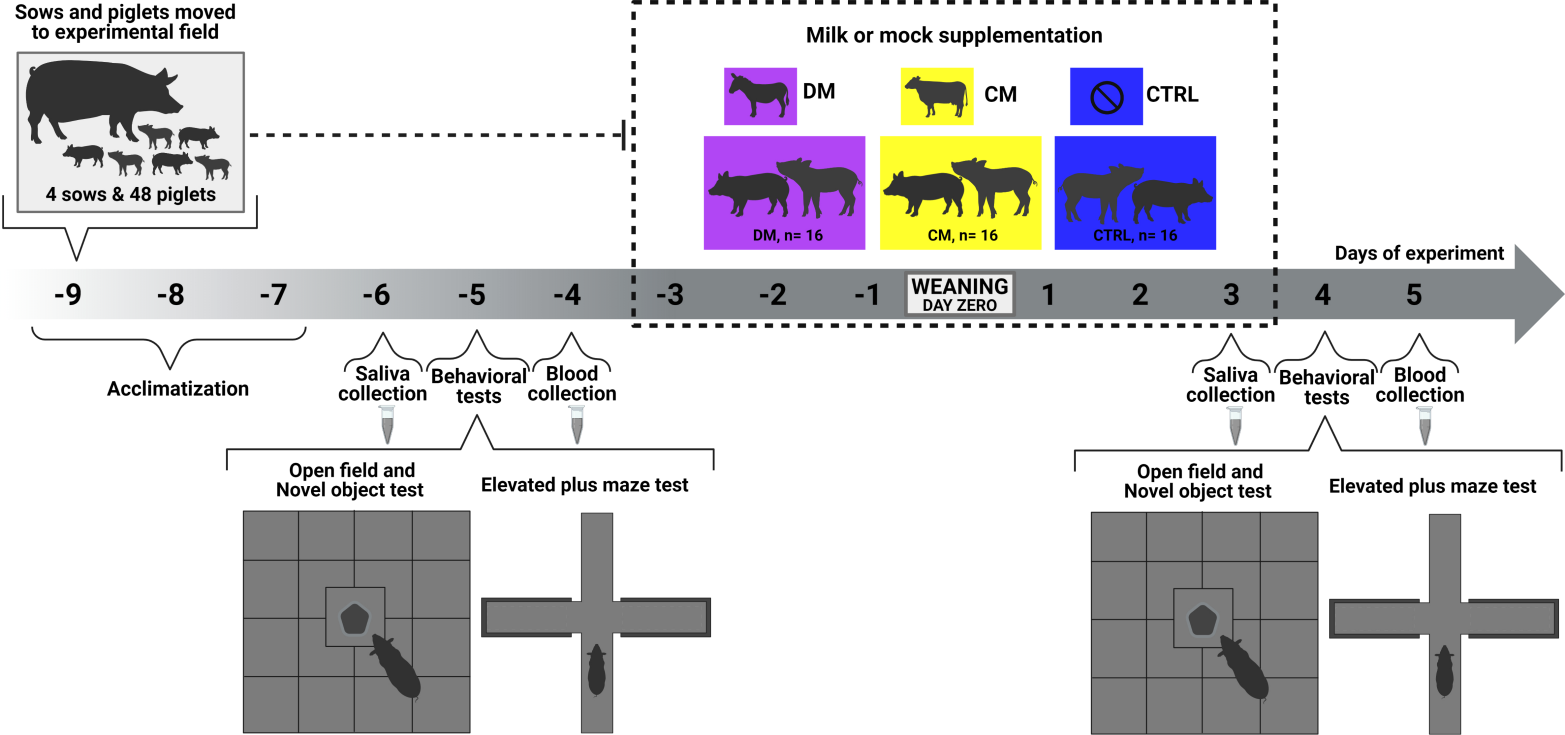
Experimental scheme showing a detailed timeline to assess asinine milk (DM) and skimmed cow milk supplementation (CM) effect on piglets’ behavior, salivary cortisol concentration, and gene expression before and after weaning. Control group (CTRL).

The experimental design was organized into blocks with four sows and litters in the pre- and postweaning phases (**Figure 1**). In each litter, four piglets each were assigned to the asinine milk supplementation group (group DM, n = 16), the skimmed cow milk supplementation group (group CM, n = 16) and the control group, with no supplementation (group CTRL, n = 16) so that all supplementation groups were represented in all litters. Piglets in groups DM and CM were supplemented for 3 days before weaning and 3 days after weaning. Before the supplementation period began, all piglets were offered nursing bottles with sugary water to test their acceptability and to get the animals used to the bottles that would later be used to offer the supplementation. Piglets that had better affinity for the bottles were randomly distributed between groups DM and CM.

The total volume of milk supplementation per day offered was a constant daily 300 mL for all piglets in groups DM and CM. During preweaning, 50 mL was offered six times a day and 100 mL three times a day postweaning. The bottles were provided to each piglet until they either drank the entire content or stopped showing interest in it. The volume of milk left in each bottle after each supplementation was noted to calculate the individual milk intake per piglet.

In the three supplementation days preweaning, the sows were removed from the pens during the supplementation period, as they did not respond well to the constant presence of the experiment team inside the pens and sometimes attempted to drink from the bottles themselves. The sows were kept together in a grass field adjacent to the pens, with access to shade, mud, food, and water, and could maintain visual, auditory, and limited physical contact with the piglets through the mesh pen doors.

The estimated nutritional value of the asinine milk and skimmed cow milk is shown in **Table 1**. The volume of milk offered to the piglets was determined according to previous studies (90), which reported that the average milk intake for piglets is between 43.1 mL/nursing at two weeks and 43.9 mL/nursing at four weeks of age. The volume of milk for each preweaning supplementation was set at 50 mL to mimic a natural nursing session.

**Table 1:** Means of nutritional values ​​found in samples of asinine milk and skimmed bovine milk used to supplement piglets before and after weaning.

Components	Skimmed bovine milk	Asinine
Proteins (% m/m)	2.9	2.94
Total fat (% m/m)	0.5	0.36
Lactose (% m/m)	4.3	4.41
Non-fat solids (% m/m)	8.4	8.00
Total Solids (% m/m)	*	8.36
Titratable acidity (lactic acid/100 mL)	0.14 -0.18	0.17
Mineral Fraction (%)	*	0.65

The “*” symbol that means the absence of data.

### 2.4 Experimental parameters

#### 2.4.1 Salivary cortisol measurement

Saliva was sampled using cotton rolls attached to dental floss to assess salivary cortisol from piglets. Sampling occurred one day before the start of milk supplementation and again on the last supplementation and was carried out at 7h am and 5h pm on both days. Cotton rolls were offered for the piglets to chew on, and once soaked with saliva, they were removed from the piglets and placed in individually identified 15-mL Falcon tubes. During sampling, the tubes were kept in polystyrene boxes lined with reusable gel ice packs and stored at –20°C. The cotton rolls were centrifuged to extract the saliva, and salivary cortisol concentrations were determined *via* enzyme-linked immunosorbent assay (91, 92).

#### 2.4.2 Behavioral tests

##### 2.4.2.1 Open field and novel object tests

To assess behavior, the open field and novel object tests were used as previously described (93, 94), aiming to determine the levels of fear and exploratory motivation in the piglets. All piglets were tested two days before supplementation began and again the day after supplementation ended. The tests were carried out at 8h am with a combination of an open field and novel object test (lasting 10 min) immediately after the piglet was introduced to the elevated plus maze test (lasting 5 min) on both days.

**Figure 2:**
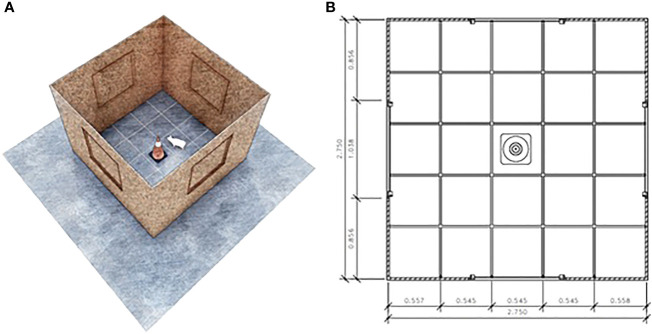
Open field and Novel object test arena **(A)**. The test arena measured 2.75 m x 2.75 m and had markings along the floor dividing the area into 25 similar quadrants **(B)**.

On each testing day, piglets were tested in a random order, and they were brought one at a time from their pens to the test arena within 30 seconds. Piglets were tested individually and placed inside the experimental arena (**Figure 2A**) for 10 minutes. The first 5 minutes inside the arena were the open field test, and afterward, an object was lowered from the ceiling through a pulley system for the 5 minutes of the novel object test (**Figure 2B**). The object used was a yellow bottle for the preweaning tests and a pink plushie for the postweaning tests, so that they would not recognize or remember the object from the previous test. All tests were recorded by a camera placed in the ceiling directly above the center of the arena, and the recordings were later analyzed to assess the behavior of each animal according to an ethogram (**Table 2**).

**Table 2:** Definitions of behaviors assessed in the Open field and Novel object tests.

Behavior	Definition
Jumping against wall	Piglet jumps against the walls of the arena.
Moving	Piglet walks in the arena.
Walking in center	Number of times the piglet crosses the center of the arena.
Time in center	Time spent by piglet in the central quadrants of the arena.
Time in edges	Time spent by piglet in the edge quadrants of the arena.
Time in the walls	Time spent by piglet to close to the walls of the arena.
Latency	Total time spent before piglet interacts with the object.
Interacting with object	Piglet interacts with the object.
Elimination	Piglet urinates or defecates.

Source: adapted from (68, 94, 95).

##### 2.4.2.2 Elevated plus maze test

**Figure 3:**
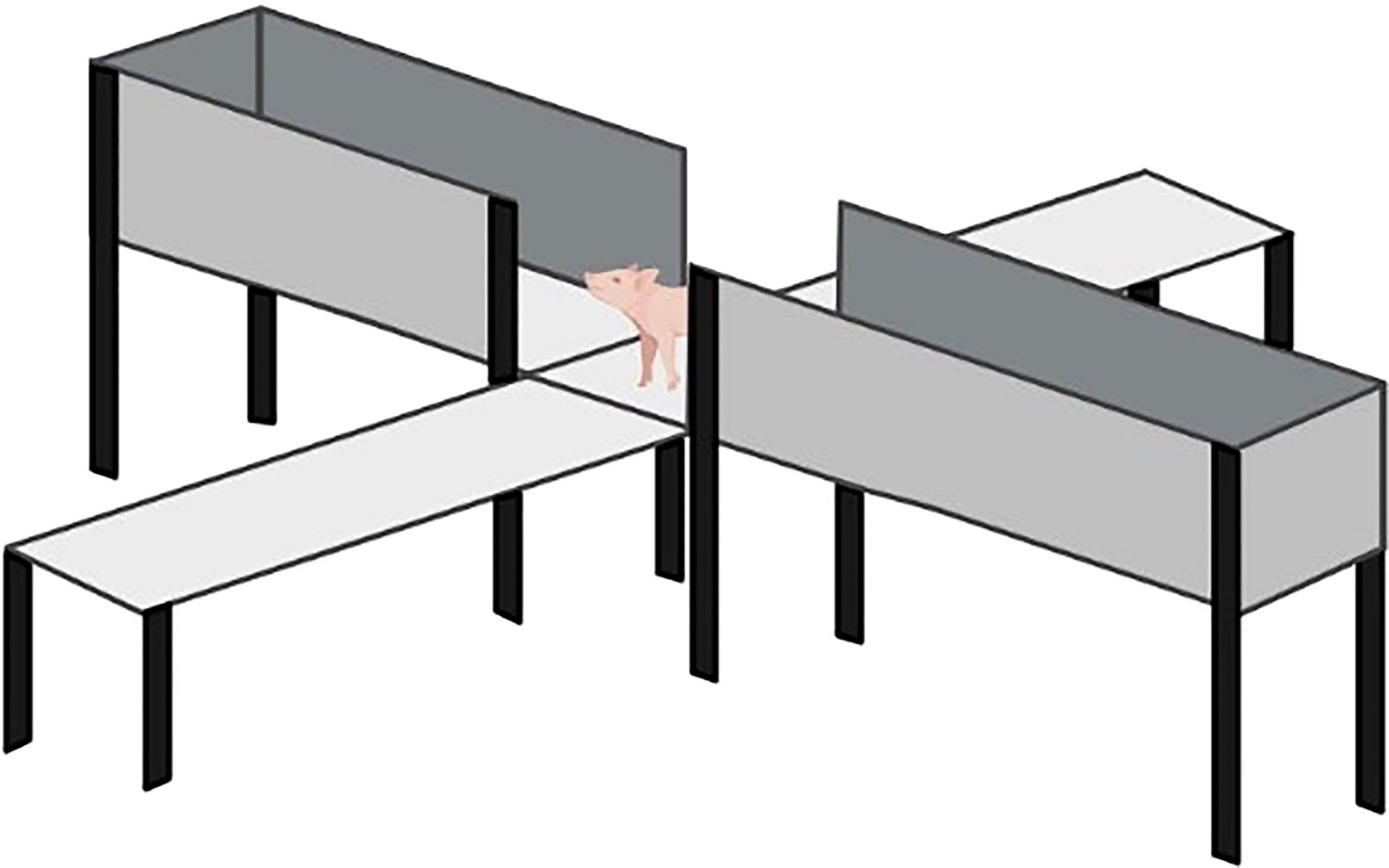
Elevated plus maze test adapted (95). The elevated measured 1.2 m long, 0.6 m wide and 0.45 m high.

The Elevated plus maze test (95) was placed in a room and surrounded by blue tarpaulin curtains so that piglets in the test could not see outside of the room. The maze was raised 1 m above the ground and had four arms of equal length and width (1.2 m and 0.6 m), and two opposite arms had walls of equal height (0.45 m) (**Figure 3**). There were rubber mats on the floor along the maze arms to break the fall of any jumping piglets.

Immediately after the open field and novel object tests, piglets were placed individually in the center of the maze and left for 5 minutes. The maze was sanitized between piglets to remove traces of urine or feces. A camera placed above the maze recorded all tests for subsequent behavior analyses.

The behaviors analyzed in the maze test were based on previously published works (95, 96), and included the time spent in the center, time spent in the open arms, time spent in the walled arms, time standing still or exploring each arm, urinating, defecating, escape attempts and jumping.

#### 2.4.3 mRNA expression assays

##### 2.4.3.1 Blood sampling

Blood sampling from the piglets was carried out on the day before the start of supplementation and two days after the end of supplementation, always starting at 7h am. The order of blood piglets sampling was randomized on all days. The blood collection protocol was based on Moreno (97) and was done from the jugular vein. Approximately 8 mL of blood was collected from each piglet from the jugular vein. Blood samples were placed into polypropylene tubes containing EDTA solution, which were immediately placed on ice and subsequently centrifuged at 2000 × g for 15 min at 4°C. Plasma was then stored at -20°C until analysis.

##### 2.4.3.2 RNA isolation and extraction

Immediately after collection, PBMCs were isolated by Ficoll^®^ gradient (Ficoll-Paque Plus, GE Healthcare, Chicago, IL, USA) using the methodology described in Pugliesi et al. (86). Briefly, whole blood was mixed with an equal volume of PBS, and the solution was layered onto 15 mL of Ficoll-Paque^®^ solution and centrifuged. After centrifugation, PBMCs were washed with hypotonic distilled water and lysis solution until a clean pellet was obtained. The remaining pellet was stored at –80°C in RNase-free tubes until RNA extraction. The purity of PBMCs was checked immediately after the procedure by staining fresh isolate samples with the quick panoptic protocol according to the manufacturer’s instructions (**Table 3**). Samples were considered pure when 95% of the 200 counted cells were polymorphonuclear cells.

**Table 3:** Swine specific oligonucleotide forward (F) and reverse (R) primer sequence (5’-3’), amplicon length of the evaluated genes, and primer efficiency in the standard curve on qPCR.

Variable in time	Phase	PC1 (%)	PC2 (%)	Total
In edges	Before	59.0	1.10	60.1
Close to the walls		30.5	36.9	67.4
Central region		10.5	62.0	72.4
In edges	After	7.60	61.8	69.4
Close to the walls		61.4	2.30	63.7
Central region		31.0	35.9	66.9

##### 2.4.3.3 RNA extraction and cDNA synthesis

The isolated PBMC samples were thawed on ice, and RNA extraction was performed using TRIzol™ reagent (Thermo Fisher Scientific) according to the manufacturer’s instructions. TRIzol (1 mL) was added to each sample, and the pellets were dissolved by vortexing the tubes for 2–5 min. After a 5-min incubation at room temperature, 200 µL of chloroform was added to the samples, followed by vortexing and then a 2-min incubation at room temperature. The samples were then centrifuged at 12,000 × g for 15 min at 4°C, and the supernatant was transferred to another tube. After that, 500 µL of isopropyl alcohol was added to each sample, followed by vortexing and incubating at room temperature for 10 min. The samples were then centrifuged at 12,000 × g for 15 min at 4°C. One milliliter of 75% ethanol was added to each sample and centrifuged at 7500 × g for 5 min at 4°C. The supernatant was removed, and the remaining pellet was stored at –80°C.

Total RNA samples from PBMCs were treated with DNAse I (Life Technologies, Carlsbad, USA) for 15 min at room temperature in a 10-μL reaction volume. The concentration of total RNA extracts was measured using a spectrophotometer (NanoVue, GE Healthcare, Chicago, USA). The isolated RNA (1.0 μg) was subjected to reverse transcription (High-Capacity cDNA Reverse Transcription Kit; Life Technologies) according to the manufacturer’s instructions, and the cDNA of each sample was stored at –20°C until qPCR analysis.

##### 2.4.3.4 cDNA synthesis and real-time PCR (qPCR)

Quantification of specific transcripts was performed by real-time polymerase chain reaction (RT−qPCR) using SYBR Green (Life Technologies, Carlsbad, CA, USA), and the reactions were carried out using a Step One Plus apparatus (Life Technologies). The mRNA abundance of the target genes *IL1B*, *HSD1B2*, and *NR3C1* was quantified by quantitative reverse transcription PCR and normalized in relation to the reference genes *(GAPDH* and *UBB).* The transcripts were selected according to Silva et al. (98) and the primer sequences are described in **Tables 3**, **4**.

**Table 4:** Swine specific oligonucleotide forward (F) and reverse (R) primer sequence (5**’**-3**’**), amplicon length of the evaluated genes, and primer efficiency in the standard curve on qPCR.

Target name	GenBank ID	Primer (5’-3’)		Amplicon	Efficiency (%)
*HSD11B2*	AF414125	F:5’ GCGAAAGCTTCCCACTGAAC 3’		59 bp	102.63
		R: 5’ AGGGTCTGTTTGGGCTCATG 3’			
*NR3C1*	AF141371	F: 5’ GATCATGACCGCACTCAACATG 3’		68 bp	97.11
		R: 5’ TTGCCTTTGCCCATTTCAC 3’			
*IL1B*	XM_021085847.1	F: 5’ TTTGAAGAAGAGCCCATCATCC 3’		119 bp	97.98
		R: 5’ CCAGCCAGCACTAGAGATTTG 3’			
*GAPDH*	NM_001206359.1	F: 5´ TCCTGGGCTACACTGAGGAC 3´		123 bp	109.59
		R: 5´ ACCAGGAAATGAGCTTGACG 3´			
*UBB*	U72496.1	F: 5´ ACCAGCAGCGTCTGATTTTT 3´		92 bp	100.03
		R: 5´ CAAGTGCAGGGTGGACTCTT 3´			

The synthesized cDNA products were used as the template for real-time polymerase chain reaction (RT−PCR) amplification (Applied Biosystems, California, USA). The reactions were run in triplicate on a 96-well plate, which was sealed with a MicroAmp optical adhesive cover (Life Technologies) before reading. The thermocycling profile consisted of 40 cycles of 15 s at 95°C for denaturation and 12 s at 60°C for annealing and extension, including a previous activation step of 95°C for 10 min. The final stage included an analysis of the melting curve, verifying the presence of a single peak in the different PCRs.

The expression of each gene was quantified by determining the threshold cycle value (CT) for the fluorescence of the SYBR green dye within the geometric region of the semilog graph generated during PCR. In the exponential phase of the amplification curve, the quantity of cDNA is considered to be duplicated in each amplification cycle. The amplification data were extracted from the Step One Plus apparatus, and each sample was analyzed through the LinReg PCR software^®^ for baseline correction, determination of qPCR efficiency and cycle quantification values (Cq). The geometric mean of the expression of these two housekeeping genes was used for normalization of the expression of the target genes. Expression of each gene relative to the expression of the housekeeping genes was normalized by the comparative Ct method corrected for amplification efficiency (99).

### 2.5 Statistical analyses

To test the effect of supplementation on the weight and consumption of piglets, a randomized block design was used with repeated measurements over time by the PROC MIXED command of the SAS Statistical Package (software version 9.3). Comparisons between means were made by Fisher’s test. For all data, significance was declared when p≤ 0.05.

In order to test the effect of supplementation on the weight and milk consumption of piglets, a randomized block design was used with repeated measurements over time by the PROC MIXED command of the SAS Statistical Package (software version 9.3). Comparisons between means were made by Fisher’s test. For all data, significance was declared when p≤ 0.05.

Regarding salivary cortisol, the weaning effect was calculated as the ratio between the salivary cortisol concentrations of piglets before and after weaning, considering the collection period (morning (AM) or afternoon (PM) (morning ratio = before weaning AM/after weaning AM; afternoon ratio = before weaning PM/after weaning PM). The effect of the collection period was determined using the ratio between the morning and afternoon salivary cortisol concentrations of piglets, both before and after the weaning event (ratio before weaning = AM before weaning/PM before weaning; ratio after weaning = AM after weaning/PM after weaning).

Grubbs’ test was performed to determine the presence of outliers in the raw salivary cortisol values considering the collection period (morning and afternoon) and the weaning event (before and after weaning).

Data distribution was determined using the Shapiro−Wilk test. Subsequently, a nonparametric Kruskal−Wallis and Nemenyi tests were performed to compare the ratios mentioned previously between the supplementation groups. The significance level for the Grubbs’, Kruskal-Wallis, and Nemenyi tests was p ≤ 0.05. Analyses were performed in R version 4.0.5 (100).

Principal component analysis (PCA) was performed to evaluate how the behavior measures explained the variance of each principal component in the open field, novel object and elevated plus maze tests in each phase using the FactoMineR (101) and factoextra (102) software packages of the R Core Team software (103).

To test the fixed effect of supplementations and phases (before and after weaning) on piglet behavior, a randomized block design was also used with repeated measurements over time with the PROC MIXED command of the Statistical Package SAS (Software version 9.3). Comparisons between means were made by the Fisher’s test was used to compare the means.

Data on gene expression that were not normally distributed according to the Shapiro–Wilk test were transformed to natural logarithms. Gene expression was analyzed using analysis of variance (ANOVA) with repeated measures over time using the PROC MIXED command of the SAS Statistical Package (software version 9.2) (104), with animal considered a random effect and supplementation and their interaction considered fixed effects. The ratio of gene expression after and before supplementation was compared by ANOVA using the PROC MIXED command (SAS). Comparisons between groups within a day were made using the least significant difference test. The results are presented as arbitrary units.

## 3 Results

### 3.1 Body weight and milk intake

There was no effect of treatments on final body weight of piglets (p > 0.05). The descriptive statistic of milk intake and body weight is presented in **Tables 4** and **5**, respectively.

**Table 5:** Descriptive statistics of milk intake for piglets in supplementation of asinine milk (DM), skimmed cow milk (CM) and no supplementation (CTRL).

Group	Mean ± SD	Minimum	Maximum	CV (%)
DM	294.67 ± 11.69	258.30	300.00	3.96
CM	243.68 ± 77.27	102.50	300.00	31.71
CTRL	–	–	–	–

SD, standard deviation from the mean; CV, coefficient of variation.

### 3.2 Salivary cortisol

**Figure 4:**
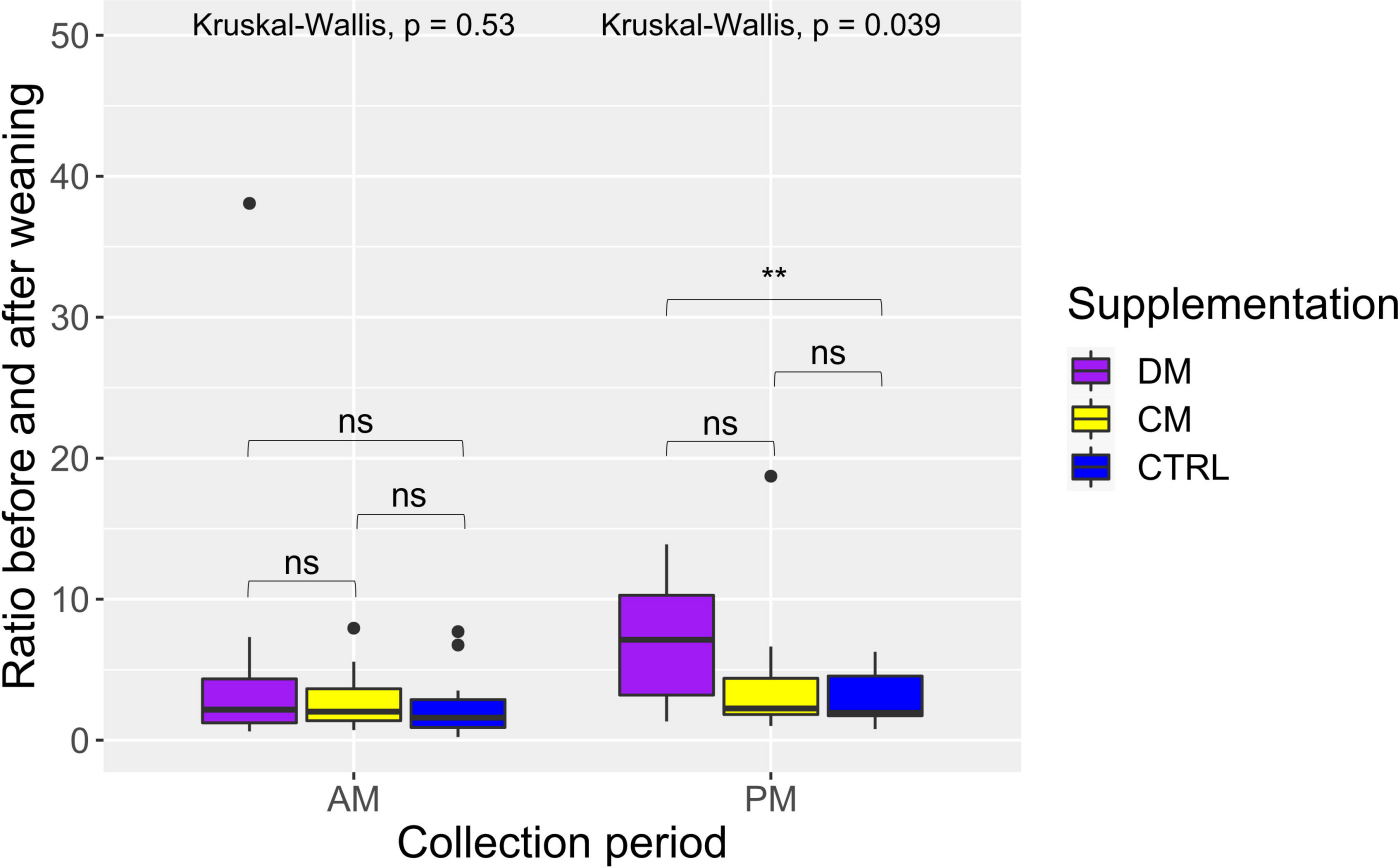
Distribution and comparison results of the salivary cortisol ratio calculated before and after weaning considering the period of collection (AM: morning and PM: afternoon) and supplementation treatment of piglets. Supplementation: DM (asinine milk); CM (skimmed cow milk); CTRL (Control without supplementation). Statistical description: ns (not significant, p > 0.05); ** (significant, p < 0.01).

Regarding salivary cortisol, 20 data points were removed from the analysis as they were considered outliers in the Grubbs test. The proportion of outliers removed per group was DM = 6.25%, CM = 12.5%, and CTRL = 12.5%. When comparing groups according to time collection (morning or afternoon) and using the weaning event for the ratio (before weaning/after weaning), there was a significant difference (p < 0.05), where piglets of group DM had a higher salivary cortisol ratio than that of group CTRL in the afternoon period (p < 0.05) (**Figure 4**). No differences were found in the remaining comparisons (p > 0.05).

### 3.3 Behavioral tests

#### 3.3.1 Open field and Novel object test

**Figure 5:**
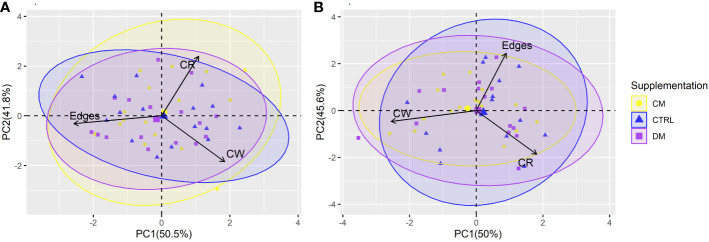
Principal component analysis (PCA) of the piglet’s behavior in the Open field test before **(A)** and after **(B)** weaning (n = 48) submitted to three supplementations: DM (Asinine milk), CM (Cow skimmed milk) and CTRL (Control without supplementation). Behaviors: Edges (time in edges), CW (time close to the walls), CR (time in the central region of the Open field test arena).

The first two PCs from the PCA of the behaviors evaluated before and after weaning explained 92.3% and 95.6% of the total variation in the data, respectively. PC1 in the phase before weaning explained 50.5% of the variation, and PC2 explained 41.8%. After weaning, values of 50% and 45.6% were observed for PC1 and PC2, respectively (**Figure 5**).

A difference was observed in the behavioral variables in PCAs between periods. Edge behavior had little contribution to PC2 before weaning but a significant contribution after weaning. In contrast, the CW behavior had little contribution to PC2 only after weaning.

The time spent in the central region (**Table 6**) was the behavioral variable that possibly best explained the total variability of the data before weaning. Most of the ellipses intersect with each other, indicating that the type of supplementation possibly did not interfere with the behavioral variables studied in the maze test.

**Table 6:** Descriptive statistics of the piglet’s body weight f (N=16) before (initial) and after (final) supplementation of asinine milk (DM), skimmed cow milk (CM) and no supplementation (CTRL).

Group	Weight	Mean ± SD	Minimum	Maximum	CV (%)
DM	Initial	7.22 ± 1.83	4.8	10.3	25.39
	Final	8.81 ± 2.66	5.8	14.0	30.27
CM	Initial	7.54 ± 1.89	5.1	10.8	25.10
	Final	9.12 ± 2.64	4.0	14.0	28.96
CTRL	Initial	6.79 ± 1.68	4.1	09.9	24.83
	Final	8.63 ± 2.56	5.0	13.5	29.67

SD, standard deviation from the mean; CV, coefficient of variation.

**Figure 6:**
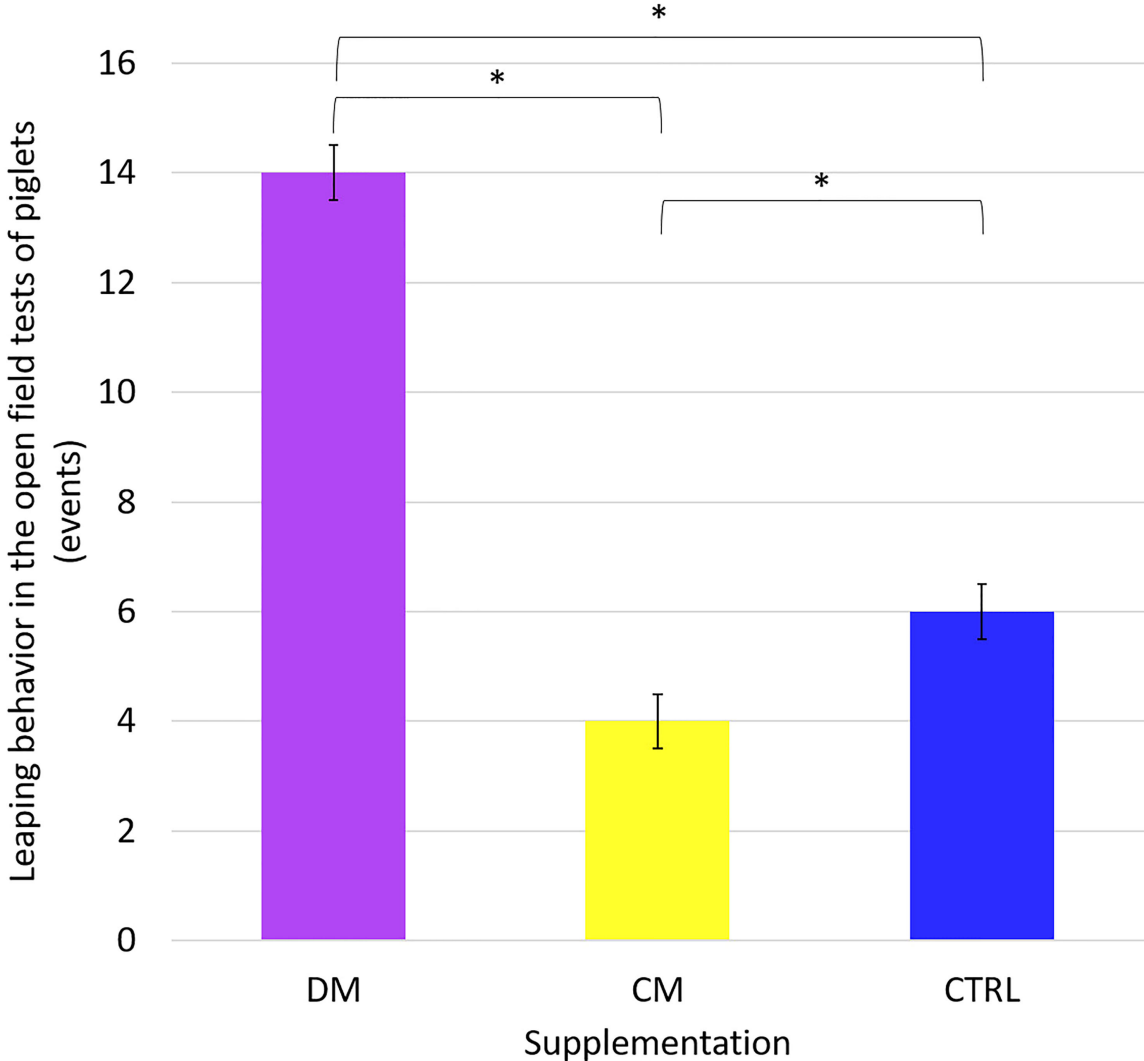
Leaping behavior in the Open field tests of piglets (n = 48) supplemented with: DM (Asinine milk), CM (Cow skimmed milk) and CTRL (Control without supplementation) in the phases before and after weaning. Statistical description: (p > 0.05); *(significant, p < 0.05) by Fisher’s test.

Furthermore, the tests of comparisons between the average pairs of the climbing the wall, jumping against the wall, and excretion behaviors as a function of supplementation were analyzed. Only the jumping against the wall behavior differed (p < 0.05) between the studied supplementation treatments (**Figure 6**). This result demonstrates that the piglets supplemented with asinine milk jumped more against the wall than the animals in the other supplementation groups before and after the supplementation phase.

Regarding the findings of the novel object test, no differences were observed in the latency (supplementation: p > 0.05; phase: p > 0.05) or interaction time (supplementation: p > 0.05; phase: p > 0.3) of piglets when considering the median values of the behavioral variables.

#### 3.3.2 Elevated plus maze

**Figure 7:**
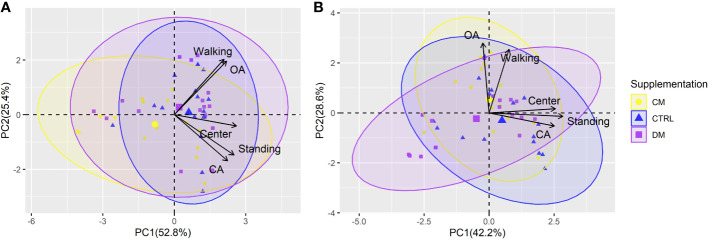
Principal component analysis (PCA) of the piglet’s behavior in the elevated plus maze test before **(A)** and after **(B)** weaning (n = 48) submitted to three supplementations: DM (Asinine milk), CM (Cow skimmed milk) and CTRL (Control without supplementation). Behaviors: OA (open arms), CA (closed arms), Center, Standing and Walking. Ellipses indicates the region with 95% confidence of the data for each treatment.

The first two PCs from the PCA referring to the behaviors evaluated before and after weaning explained 78.2% and 70.8% of the variation in the total data, respectively (**Figure 7**). Before the weaning phase, PC1 explained 52.8% of the variation, and PC2 explained 25.4%. After weaning, values of 42.2% and 28.6% were found for PC1 and PC2, respectively.

A difference was observed in the PCAs in the response of variables in relation to the pre- or postweaning period, meaning that the contribution toward data variability from certain behaviors was different. For example, the CA behavior contributed similarly to explaining the variability of PC1 and PC2 before weaning; however, it did not contribute significantly to PC2 after weaning. On the other hand, OA behavior had little participation in PC1 after weaning. The Center variable had a similar contribution in the PCA in both periods.

Walking behavior and the time spent in the open arm were the variables that explained most of the total variability in the data. The points match the PC1 and PC2 scores before and after weaning the piglets (**Table 7**). Most ellipses intersect with each other, indicating that the type of supplementation may not interfere with the behavioral variables studied in the labyrinth test.

**Table 7:** Contributions (in percentage) of the variables to the principal components (PC1 and PC2) measured Elevated plus maze test before and after weaning of piglet’s.

Variable in time	Phase	PC1 (%)	PC2 (%)	Total
Open arms	Before	17.7	29.2	46.9
Closed arms		18.3	21.7	40.0
Central region		25.0	1.30	26.3
Walking		16.0	31.3	47.3
Immobility		23.0	16.5	39.5
Open arms	After	0.27	53.5	53.8
Closed arms		29.3	1.89	31.2
Central region		30.2	0.20	30.4
Walking		2.74	44.3	47.0
Immobility		37.4	0.13	37.5

**Figure 8:**
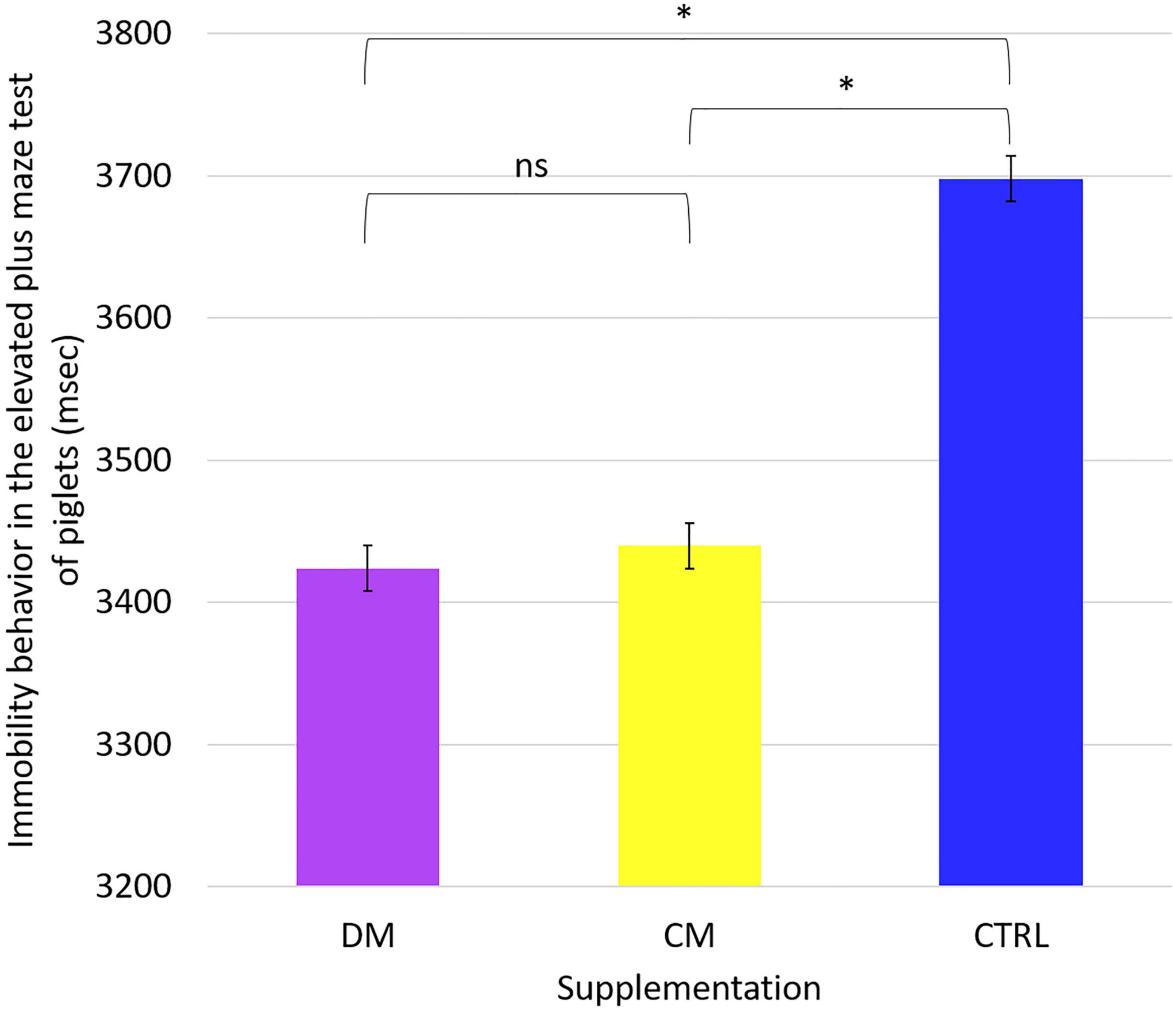
Immobility behavior in the Elevated plus maze tests of piglets (n = 48) supplemented DM (Asinine milk), CM (Cow skimmed milk) and CTRL (Control without supplementation) during the period from 25 to 31 days of life. Statistical description: ns (not significant, p > 0.05); * (significant, p < 0.05) by Fisher’s test.

We analyzed the mean values of the behavioral responses of the piglets in the maze test. We found no differences between the mean values of the time spent in the behavioral variables: walking, staying in the center, staying in the open arm, and staying in the closed arm. Only the immobility behavior differed (p < 0.05) between the groups that received supplementation compared to the control group (**Figure 8**).

This finding demonstrates that the animals in the control group performed more movements than the other supplementation groups.

### 3.4 mRNA expression assays

**Figure 9:**
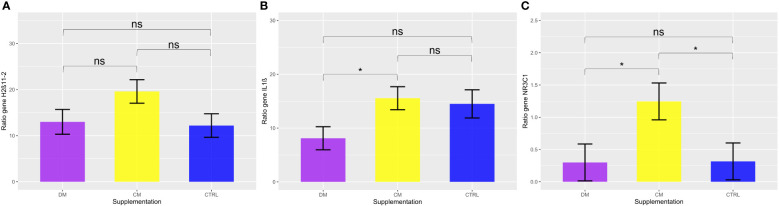
Analysis of the gene expression of the three evaluated genes **(A)** HSD11β2, **(B)** IL-1β, and **(C)** NR3C1, considering 2∆∆Ct values. Comparison between milk supplementation DM (Asinine milk), CM (Cow skimmed milk), and CTRL (Control without supplementation) in the post-weaning period. Data represent averages ± standard error of the mean (SEM). Statistical description: ns (not significant, p > 0.05); * (significant, p < 0.05).

The expression of the *IL-1B* gene (**Figure 9B**) was significantly downregulated in the group fed asinine milk (mean 8.11 ± 2.14) in relation to the group supplemented with skimmed cow milk (mean 15.55 ± 2.14) (p < 0.05). No differences when compared to expression in the control group (mean 14.50 ± 2.62; p > 0.05).

**Figure 10:**
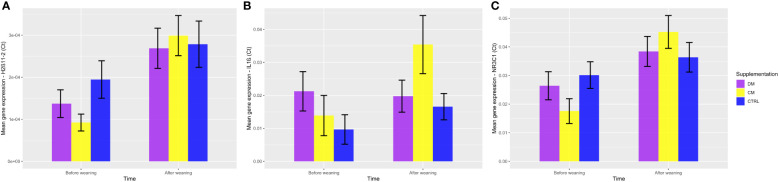
Representation of the gene expression of the three evaluated genes, **(A)** HSD11β2, **(B)** IL-1β, and **(C)** NR3C1, considering 2∆∆Ct values, divided by supplementation group and weaning event. DM (Asinine milk), CM (Cow skimmed milk), and CTRL (Control without supplementation). Data represent averages ± standard error of the mean (SEM).

When analyzing the genes related to the stress response by the HPA axis, there was significant upregulation of the *NR3C1* gene in the group supplemented with cow milk (mean 1.245 ± 0.28) when compared to the group that received asinine milk (mean 0.299 ± 0.28; p < 0.05) and the control group (mean 0.315 ± 0.28; p < 0.05) (**Figure 9C**). Additionally, no significant difference (p > 0.05) was found between the groups supplemented with asinine milk, skimmed cow milk and the control group pre- or postweaning for the expression of *HSD11B2* (**Figure 9A**). See **Figure 10** for raw genetic expression data.

## 4 Discussion

The absence of differences between groups in weight and milk intake parameters is possibly explained by the low concentration of protein and fat in both asinine milk and skimmed cow milk. The average protein level of asinine milk is 1.5% to 1.8%, and the concentration of fat is 0.2% to 1.8%, similar to human milk (75, 105, 106).

These results were expected, as supplementation with both types of milk was not meant to enhance weight gain but to assess potential changes in stress response modulation at weaning. The acceptability of both types of milk during supplementation was considered adequate.

The decrease in salivary cortisol in the afternoon period could potentially be assigned to the circadian rhythm of cortisol secretion, in which cortisol is physiologically produced at higher levels in the morning and lower levels in the afternoon (107). However, this is probably not the case in this study, as the circadian rhythm of cortisol in piglets is established at 16 to 20 weeks of age, with the afternoon decrease in cortisol levels only present after 8 weeks (108), and all piglets in this study were no older than 5 weeks old.

The lower levels of salivary cortisol postweaning in piglets supplemented with asinine milk could result from various factors of the milk composition, such as the high concentrations of lactose, PUFAs and lysozyme (109, 110) which are greater than those of cow milk. The specific composition of asinine milk could be relevant for this result, as previous studies have elucidated valuable information. For example, researchers have demonstrated that lysozyme in asinine milk represents 21% of the protein fraction (111, 112). This enzyme is poorly digested in the gastrointestinal tract and acts primarily at the gut level in microbiota modulation, favoring healthy compositions (113–115). In addition, microbiota changes may influence the gut-brain axis (116, 117), potentially protecting piglets supplemented with asinine milk from harmful effects caused by weaning stress.

Another factor that was previously explored involves the fat profile of asinine milk (118–121). Asinine milk supplementation provided higher PUFA levels compared to cow’s milk, and these compounds may also exert modulatory effects on stress responses (122), having been found to positively alter the stress response of piglets during weaning (123).

Asinine milk is also rich in lactose, which is an important source of galactose. This carbohydrate is a key structural element in complex molecules that are crucial for early development (124), and may also positively alter the gut microbiota toward profiles that are compatible with favorable stress responses (125). Lactose may also influence acceptability, especially in children, explaining why asinine milk is considered highly palatable (126–128). The ingestion of palatable foods may reduce stress levels and have mood-altering effects (129–132) that may have contributed to the lower stress response in piglets fed with asinine milk.

Moreover, asinine milk oligosaccharides may have the capacity to modulate the proliferation, apoptosis, and differentiation of intestinal cells (133, 134) and may assist in brain development and cognition (76, 124), by being playing a role in the formation of myelin (++3) and providing sialic acid (++8 ++9) (75, 135). Nevertheless, additional research utilizing longer supplementation periods, higher milk volumes and different types of milk are needed to assess whether they have modulating effects on systemic events that constitute stress responses. As in this study, the supplementation time was only 6 days, so there might not have been enough time to generate greater modulatory effects compatible with the broad range of health benefits potentially offered by asinine milk.

Although the salivary cortisol assessments indicate that piglets fed with asinine milk were less intensely stressed than those in the other groups, the results from the behavioral tests do not reflect that finding. All groups performed similarly in the behavioral tests regarding indicators of fear and anxiety, with the exception of a single variable: piglets supplemented with asinine milk jumped against the walls more during the open field/novel object tests in the postweaning assessments than piglets in the other supplementation groups did in the same period. It is not possible to tell from this single behavioral variable whether these animals were more or less stressed than the others.

It is known that in emotional tests, the most basic and common result of interest is “movement”; however, this can be influenced by motor output, exploratory drive, freezing, or other behaviors related to fear, illness, and relative timing in the circadian cycle, among many other variables (69).

The high concentration of PUFAs in asinine milk, compared to the other supplementations, could potentially have generated greater behavioral effects in these piglets, as demonstrated by the supplementation of medium- and long-chain PUFAs and the modulation of behavior in piglets in other studies (136).

Another important factor for consideration is that the behavioral tests in this study were conducted both pre- and postweaning; therefore, the repetition could have influenced the reaction of all piglets in the second exposure to the arenas. Animals exposed to the open field tests a second time tend to be less active and less explorative and produce fewer vocalizations than those in their first exposure to the arena (137).

Other studies using asinine milk to supplement piglets are unknown, and the use of behavioral measurements to assess the physiological significance of early changes in the diet on the developing pig brain is not yet well established (136). Further studies with varying supplementation times and volumes are needed to understand the possible modulation mechanisms of the physiological and behavioral responses of piglets to stress. The inclusion of asinine milk for a longer period could be a suitable option, as other studies have found benefits when supplementing piglets with PUFAs for more than 30 days (138–140).

Regarding the gene expression profiles in response to weaning, no significant difference in the expression of the *11β-HSD2* enzyme, which converts cortisol to the inactive form cortisone (141) was observed, but we did find a significant upregulation in expression of the *NR3C1* gene at postweaning in the piglets fed skimmed cow milk when compared to both of the other supplementation groups. In the assessment of salivary cortisol levels, we found that piglets supplemented with asinine milk had lower cortisol levels postweaning in the afternoon samples when compared to both of the other supplementation groups in the same period.

The upregulation of *NR3C1* expression, as observed after weaning in piglets fed skimmed cow milk, has been noted in animals that experienced early life stress (142) and in humans diagnosed with posttraumatic stress disorder (PTSD) (143, 144). However, this result contradicts the findings of studies on weanling piglets (54), that reported downregulation of *NR3C1* expression in contrast to high levels of circulating cortisol.

Research on the dynamics of glucocorticoid receptor expression in response to stress is a relatively new field (145, 146), and further studies are needed to elucidate the relevance and nature of glucocorticoids in relation to *NR3C1* expression (143, 144). Moreover, the present results refer to gene transcription, as they reflect mRNA levels, and may not reflect the effective protein expression of glucocorticoid receptors or their density in the central nervous system.

The results of the mRNA expression of *NR3C1* did not correspond to the levels of salivary cortisol from the piglets, as there were no significant alterations in relation to salivary cortisol levels in piglets supplemented with skimmed cow milk. Instead, the group of piglets supplemented with asinine milk presented lower cortisol levels in the afternoon postweaning in comparison to both of the other supplementation groups in the same period. We suggest that these animals experienced a less intense stress response in this period than that of the piglets fed with skimmed cow milk and the control group, as the exposure to stressors such as weaning causes significant activation of the HPA axis, as evidenced by the greater production of glucocorticoids (147–149).

The significant variation in *IL-1B* expression profiles between the experimental groups can probably be attributed to the fact that each supplemental milk used in the study possesses different nutritional properties (73, 124, 150), which favored varying intensities of immune reaction in response to the weaning challenge.

The downregulation of *IL-1B* expression in PBMCs of piglets fed asinine milk follows patterns observed in rats that received asinine milk for 4 weeks, in which these animals presented with lower serum concentrations of IL-1 and TNF-α (119, 151) and a lower inflammatory state in muscle tissue when compared to those of rats that received cow milk or no supplementation. These results differ from the findings of a study in which asinine milk was offered to elderly humans, who showed increased levels of plasma *IL-1B*, *IL-8* and *IL-6* (88); however, the latter results were beneficial in the specific context, as the subjects were immunocompromised. Additionally, when asinine colostrum and milk were added to human PBMCs, they showed the potential to modulate the expression of *IL-1B*, *TNF-α*, *IL-10*, and *IL-12* (88, 152).

The fat profile of asinine milk, which contains high concentrations of PUFAs (124), might have influenced the downregulation of *IL-1B* expression observed in this study, as these fatty acids are generally considered anti-inflammatory (153–155). Moreover, the lactoferrin content of asinine milk may also have contributed to this result, as the supplementation of lactoferrin in suckling piglets for 7 days has been previously associated with a decrease in *IL-1B* and *TNF-α* and an increase in *IL-10* concentrations in the intestinal mucosa, favoring an anti-inflammatory profile (156).

On the other hand, the upregulation of *IL-1B* expression in the group fed skimmed cow milk might be related to its lipid composition, as it contains higher concentrations of saturated fatty acids (SFAs), which may favor proinflammatory responses (151), and poor concentrations of bioactive molecules such as lactoferrin (84), which does not contain lysozyme.

In addition to cytokines, immunoglobulins could also have been used in this study as a parameter of the immune response during weaning stress. The concentration of IgA in piglet saliva may be modulated by stressful situations, reaching values of 500 mg/L to 800 mg/L from basal concentrations of 100 mg/L in the presence of stress. Future studies may explore the interaction between asinine milk supplementation and the IgA response during a stress challenge, such as the weaning period.

Further studies are needed to investigate whether the inclusion of asinine milk represents an adequate strategy to mitigate the negative effects of stressful events in early life. Future studies should consider improvements in the experimental design, such as supplementation times and concentrations, to determine the minimum periods and volumes needed that could provide benefits in relation to the stress response. Studies with varying amounts of asinine milk and using a larger sample size are needed to better answer the questions raised. Our study showed that piglets that received asinine milk presented lower postweaning blood cortisol levels; however, further studies are needed to elucidate the molecular mechanisms involved in the production, secretion and the receptors involved in this physiological axis, since our data were inconclusive.

## 5 Conclusion

Supplementation with asinine milk modulates the increase in salivary cortisol levels of piglets undergoing the stress of weaning and may have the potential to improve immunity parameters without affecting the expressive behavioral response. Therefore, asinine milk supplementation may benefit human infants, and further research should explore this possibility. Further studies should investigate the mechanisms behind the alterations found in cytokine gene expression and cortisol concentrations, as well as the changes in other molecules that may be mediated by asinine milk supplementation.

## Data availability statement

The original contributions presented in the study are included in the article/supplementary material. Further inquiries can be directed to the corresponding author.

## Ethics statement

The animal study was reviewed and approved by Committee for the Use and Care of Animals in Research (CEUA) of the School of Veterinary Medicine and Animal Science of the University of São Paulo, protocol n° 8696141117 (ID 007216).

## Author contributions

SF and AZ contributed to the study conceptualization. SF, CO, and AZ designed the methodology. SF, LS, AN, PF, and GP realized the data validation. MS, GP, CR, and CO performed the statistical analysis. SF, AD, VM, LS, and AN acquired and organized the data. SF, AD, and AN interpreted the data. AZ provided the resources. SF, AD, AN, and PF contributed to the writing-original draft preparation. SF, AD, VM, LS, MS, AN, PF, GP, CR, CO, and AZ worked on the writing-review and editing. CO and AZ supervised the study. All authors contributed to manuscript revision and read and approved the submitted version. All authors contributed to the article and approved the submitted version.
